# A retrospective review of group B streptococcus bacteraemia in Western Sydney, Australia from 2011-2023

**DOI:** 10.1016/j.ijregi.2025.100610

**Published:** 2025-02-26

**Authors:** Jimmy Shen, Ravindra Dotel, Clinton M.G. Colaco

**Affiliations:** 1 The University of Sydney, Westmead Clinical School, Sydney, Australia; 2 Department of Infectious Diseases, Blacktown Hospital, Sydney, Australia; 3 Centre for Infectious Diseases and Microbiology, Westmead Hospital, Sydney, Australia; 4 Rural Clinical School, University of Queensland Faculty of Medicine and Biomedical Sciences, Rockhampton, Australia

**Keywords:** Group B streptococcus, Bacteraemia, Epidemiology, Clinical manifestation, Antibiotics, Streptococcus agalactiae, Retrospective review, Sydney, Australia

## Abstract

**Objectives:**

This study aimed to describe the epidemiology, clinical manifestations, management, and outcomes of group B streptococcus (GBS) bacteraemia in a large cohort spanning 13 years.

**Methods:**

This retrospective study included patients aged 16 years or older with positive GBS blood cultures obtained from four metropolitan hospitals in Western Sydney, Australia, from 1 January 2011 to 31 December 2023, inclusive. The case details of these positive cultures were reviewed using electronic medical records.

**Results:**

A total of 344 patients with GBS bacteraemia were included during the 13-year study period. The median age was 65 years, and 50% of the patients were aged 65 years or older. Known risk factors were identified in 83.1% of the patients with cardiac disease, and diabetes mellitus was the most prevalent. Skin and soft tissue infections were the most common primary manifestation (32.0%). Pregnancy-related infections represented a notable proportion of the cases (16.3%). The 7- and 30-day mortality rates were 2.3% and 5.2% respectively, with a higher mortality rate of 9.3% for those aged 65 years or older. Penicillins were the most common antibiotic class used to treat GBS bacteraemia and its resultant manifestations (66.1%).

**Conclusions:**

GBS bacteraemia most commonly occurs in individuals aged ≥65 years or during pregnancy. While mortality rates are generally low, they are twice as high in those aged ≥65 years.

## Highlights

• Morbidity and mortality of group B streptococcal bacteraemia are linked to age.
• A significant proportion of cases have comorbid diabetes mellitus.
• Skin and soft tissue infections are a common manifestation.
• Those with endocarditis have the highest rates of mortality.
• Vaccine trials are needed for future prevention of disease.

## Introduction

Group B streptococcus (GBS), also known as *Streptococcus agalactiae*, is a leading cause of both neonatal and pregnancy-related disease [1], as well as invasive infections in non-pregnant individuals, leading to a variety of manifestations [2,3]. Skin and soft tissue infections (SSTIs), including cellulitis, are relatively common manifestations in adults, whilst less common manifestations include bacteraemia, infective endocarditis (IE), meningitis, bone and joint infections (BJIs), and urosepsis.

Known risk factors for GBS bacteraemia in adults include obesity, diabetes, immunocompromised conditions, older age (≥65 years), and pregnancy [2,3]. Conditions of the heart such as rheumatic heart disease and valve replacement may be associated with increased risk of GBS IE [3]. In addition, alcohol misuse, liver disease, kidney disease, and neurological disorders are associated with increased propensity for acquiring invasive disease [3]. The presence of comorbid risk factors significantly increases susceptibility to GBS disease, with up to 95% of cases having at least one of the aforementioned underlying conditions [2]. Obesity is becoming increasingly prevalent in Australia [4], which may affect the prevalence of GBS infections. Many of the predispositions associated with invasive GBS infection appear to ultimately stem from modifiable lifestyle risk factors [2].

Antibiotic therapy is currently the mainstay of treatment for GBS infections. Penicillin remains the antibiotic of choice. Whilst GBS is susceptible to penicillins [5], there are concerns regarding antimicrobial resistance developing locally and globally. Studies from Australia indicate resistance rates to erythromycin and clindamycin to be between 32% and 45% [5,6]. This is similar to findings from international studies from the United States [2] and the Middle East, which reported resistance to erythromycin of up to 57% and to clindamycin of up to 43% [7].

The overall 30-day mortality rate for GBS bacteraemia in non-pregnant adults has been reported to be between 6.5% and 20% [[8], [9], [10]]. This study aimed to understand the local epidemiology of GBS disease, compare the findings with other similar studies from the international community, identify areas that may need further research, and ultimately provide insight into possible interventions and comparators for future randomised controlled trials on the management and/or prevention of GBS disease.

### Aims

1. To describe the clinical epidemiology of GBS bacteraemia in a large cohort spanning 13 years (2011-2023 [inclusive]).
2. To describe the contemporary management and outcomes of GBS disease in a developed nation.

## Methods

### *Participants and study design*

We accessed the Centre for Infectious Diseases and Microbiology (CIDM) laboratory information system database of all bacteraemia cases in the Western Sydney Local Health District (WSLHD). We assessed the cases on the basis of their blood culture isolates and included them according to the following criteria.

#### Inclusion criteria

• Any blood culture with confirmed GBS isolates, collected at Auburn, Blacktown, Mount Druitt, or Westmead hospitals between 1 January 2011 and 31 December 2023, inclusive
• Age ≥16 years

#### Exclusion criteria

• Patients with polymicrobial bacteraemia in the initial blood culture, in which other bacteria were deemed significant and hence treated by clinicians, were excluded. For instance, polymicrobial bacteraemia with GBS and *Staphylococcus aureus*, in which clinicians treated both GBS and *S. aureus*, were excluded.

### *Data collection*

We retrospectively reviewed the admission details of each patient by assessing their electronic medical records (eMR). Demographics, such as age, gender, country of birth/ethnicity, principal and additional manifestation(s), investigations, antibiotic therapy (grouped by class), and duration were recorded.

Outcomes of 7- and 30-day mortality from the first GBS blood culture, intensive care unit (ICU) admission requirements, and length of stay (LOS) were recorded. To calculate the LOS, the dates of admission and discharge were determined from eMR notes or recorded encounter dates if the former was unavailable. Patients ‘admitted’ to the hospital-in-the-home service were recorded as inpatients. Transfers between hospitals and units in the WSLHD were not regarded as discharges, whereas transfers to facilities outside the WSLHD were regarded as discharges. Death certificates and recorded dates of death were reconciled to record mortality.

Risk factors on admission such as dialysis, heart disease (cardiac failure, valvular disease, myopathy, ischaemic heart disease and arrhythmias), history of malignancy (past or current), neutropoenia, liver disease (cirrhosis, alcoholic, viral, fatty liver), alcoholism, diabetes mellitus (type I and II), and obesity (defined as body mass index [BMI] >30) were recorded as per eMR entries at the time of positive blood culture (BMI measurements up to 4 months before or after were accepted). Alcoholism, either past or present, was accepted if recorded explicitly, or it was noted that a patient consumed >10 standard drinks per week or >2 per day. Long-term care facility residence was recorded on the basis of discharge or admission notes and included group homes and aged/disability care facilities, but not correctional centres or independent living facilities.

If a follow-up blood culture was performed and negative within 28 days of the index positive blood culture, this was recorded.

Manifestations were determined to be either SSTIs (cellulitis, soft tissue abscess, myositis, thrombophlebitis), BJIs (osteomyelitis, discitis, or septic arthritis), gynaecological infections (intrapartum, peripartum, and other gynaecological organ infection), pneumonia, sepsis (without focus), urosepsis, IE, intra-abdominal infections (enteritis, colitis, cholangitis, pancreatitis, peritonitis), or central nervous system (CNS) infections (meningitis or CNS-related abscesses).

The principal manifestation was defined as the diagnosed source of GBS infection or the most prominent manifestation at the time of a positive index blood culture. Probable IE was recorded according to the treating clinician's interpretation of the modified Duke criteria for IE [11]. Additional manifestations were listed. For obstetric cases, additional data about pregnancy status, antibiotic prophylaxis, and GBS screening status were recorded.

Imaging was performed during the period of admission, which included transthoracic echocardiography (TTE), transoesophageal echocardiography (TOE), computerised tomography (CT), magnetic resonance imaging (MRI), bone scan, labelled white blood cell scan, positron emission tomography (PET), and gallium scan. Targeted scans, such as MRI and CT, were included irrespective of the focus region of the body. Progress or discharge notes needed to explicitly state imaging orders, or reports had to be available for inclusion in the analysis; if these were absent, the modality was assumed not to have been undertaken.

We recorded allergies to antibiotics (if present) and the antibiotics used. Principal antibiotics (active antibiotic[s] used for the longest duration) were recorded. Intravenous (IV) antibiotics were prioritised over oral (PO) antibiotics in recording the principal treatment. The IV and PO antibiotic durations were recorded on the basis of eMR entries and scanned medication charts. The duration of antibiotic use was recorded by calculating the difference (in days, rounded to the nearest day) between the start and finish dates (if available).

### *Data analysis*

Data were recorded in Microsoft Excel, and analysis and figure generation were performed using Excel or IBM SPSS Statistics, Version 29.0, as appropriate. The chi-square test of independence or Fisher's exact test was used, as appropriate, to determine statistically significant associations. A *P*-value of <0.05 was considered statistically significant.

## Results

### *Epidemiology*

**Figure 1:**
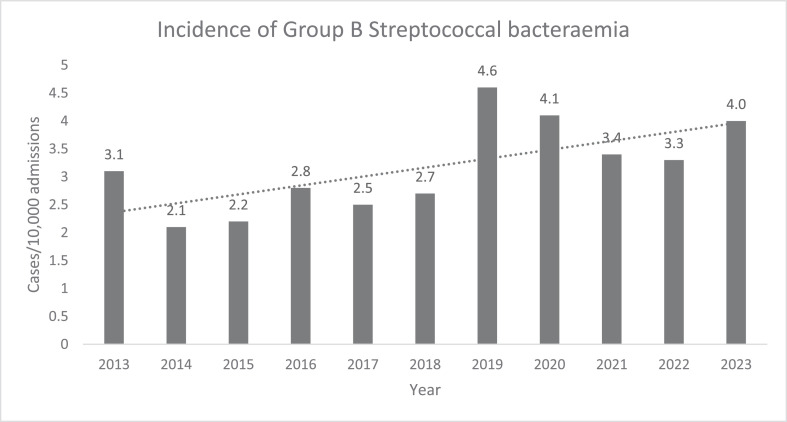
Annual incidence of group B streptococcus cases per 10,000 admissions from 2013 to 2023. Each year represented by a single column with the corresponding value atop. Trend line shown as a dotted line. Years 2011-2012 not included because of unavailability of total annual admissions data.

A total of 344 bacteraemia cases were identified during the 13-year study period. The median age was 65 years (range 17-95); 50% were aged ≥65 years, 56.1% were female, 42.2% were born in Australia, and 2.9% identified as Aboriginal and Torres Strait Islander. Of these cases, 53.5% were from Westmead Hospital (the largest of the four hospitals). The main underlying chronic diseases are outlined in Table 1a and include cardiac disease (45.6%), diabetes mellitus (43.9%), and obesity (40.7%). Two percent of patients were receiving dialysis, 10.5% resided in a long-term care facility, and 1.7% had neutropoenia. At least one risk factor (Table 1a) was identified in 83.1% of cases. The incidence of GBS per 10,000 admissions has steadily increased since 2013, as shown in Figure 1. Of the total cases, 93.9% were community-acquired.

**Table 1a:** Demographics and risk factors recorded at time of admission.

Age, y	n (%)	
16-30	33 (9.6)	
31-45	52 (15.1)	
46-60	69 (20.1)	
61-75	78 (22.7)	
≥76	112 (32.6)	
**Sex**		
Men	151 (43.9)	
Women	193 (56.1)	
**Place of birth**		
Australia - non-Aboriginal/Torres Strait Islander		145 (42.2)
Australia - Aboriginal/Torres Strait Islander		10 (2.9)
China		13 (3.8)
England		12 (3.5)
India		18 (5.2)
Lebanon		11 (3.2)
Other		128 (37.2)
Unknown		7 (2.0)
**Hospital**		
Westmead		184 (53.5)
Blacktown		114 (33.1)
Mount Druitt		34 (9.9)
Auburn		12 (3.5)
**Comorbidities and risk factors**		
Cardiac disease		157 (45.6)
Diabetes		151 (43.9)
Obesity		140 (40.7)
History of malignancy		72 (20.9)
Liver disease		40 (11.6)
Residence in long-term care facility		36 (10.5)
Alcoholism		28 (8.1)
Complication of invasive procedure		9 (2.6)
Neutropoenia		6 (1.7)
Intravenous drug use (past or present)		5 (1.5)
Dialysis		
Haemo-		5 (1.5)
Peritoneal		2 (0.6)
**Acquisition**		
Hospital		21 (6.1)
Community		323 (93.9)
**Healthcare association**		
Yes - inpatient		21 (6.1)
Yes - non-inpatient		58 (16.9)
No - community		237 (73.4)
Undetermined		28 (8.1)

Surgery- and device-related presentations comprised a large proportion of GBS bacteraemia-associated morbidity, as reported in Table 1b. SSTIs (32.0%), gynaecological infections (18.6%), and BJIs (16.9%) were the most prevalent principal manifestations, as shown in Figure 2a. Figure 2b shows all manifestations observed in the study; CNS infections, IE, and intra-abdominal manifestations each were found in 2.6% of the cases. In addition to the primary manifestations, additional manifestations were diagnosed and recorded in 25.9% of cases.

**Table 1b:** Surgery- and device-related cases.

Surgery/device		n (%)
Surgery-related		21 (6.1)
Device-related		
Prosthetic joint		5 (1.5)
Intravenous cannula		3 (0.9)
Urinary catheter^a^		3 (0.9)
Replacement cardiac valve		3 (0.9)
Central line		2 (0.6)
Permanent pacemaker or cardiac device		1 (0.3)
Haemodialysis catheter		1 (0.3)
Other		1 (0.3)

a Including suprapubic (n = 1).

**Figure 2a:**
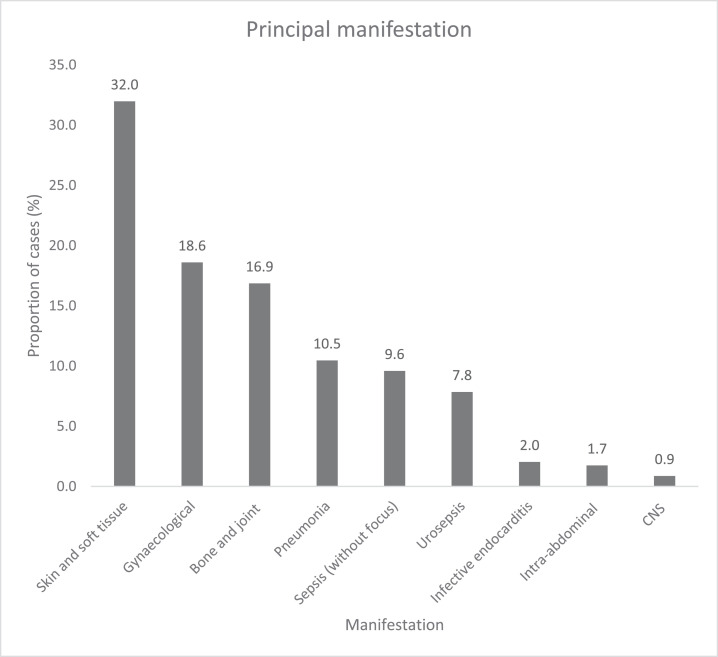
Principal manifestations. The principal manifestations of group B streptococcal bacteraemia, depicted as a percentage of the total cases (n = 344) in a bar graph in descending order. The numerical percentage is displayed atop of each bar.

**Figure 2b:**
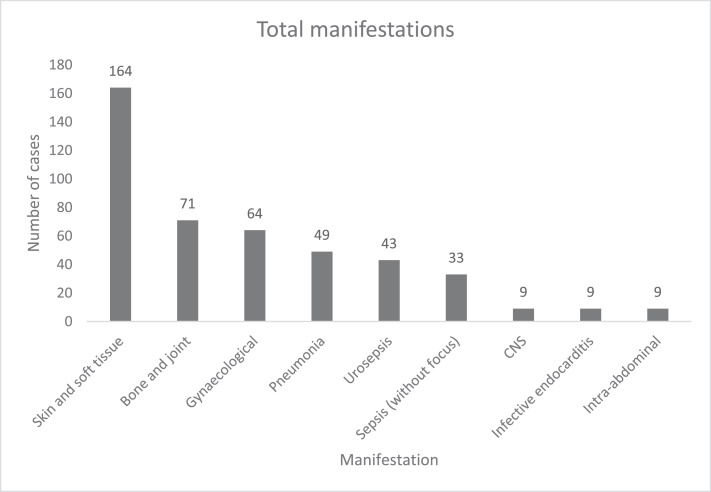
Total manifestations. Total number of manifestations of group B streptococcal bacteraemia in a column graph in descending order. Total case numbers included the principal manifestations and additional manifestations. CNS: central nervous system.

Regarding gynaecological manifestations, only 59.3% had confirmed placental/gynaecological organ cultures, and 87.5% (n = 56) were pregnancy-related (peripartum or intrapartum). Of these, 7.1% resulted in intrauterine foetal death. Among the pregnancy-related cases, 52% had GBS screening antenatally (n = 25 as part of antenatal care and n = 4 not as part of antenatal care but on admission). Prophylactic antibiotics were administered to all but nine patients, all of whom had postpartum GBS bacteraemia.

### *Outcomes*

Forty-four percent of admissions lasted 7 days or fewer, whilst the median LOS was 9 days (range 0-262). Twenty-two percent of admissions lasted 8-14 days, 14.7% lasted 15-30 days, and 18.9% lasted more than 30 days. ICU admission was required in 15.4% of the cases, whilst data for 11 cases were unavailable.

Table 2a shows all-cause 7-day and 30-day mortality according to principal manifestation. The total 7-day and 30-day mortality rates in our study were 2.3% and 5.2%, respectively. There was a significant difference in 30-day mortality between the principal manifestations (Fisher's exact test, *P* = 0.0459), but no significant difference in 7-day mortality. Of the total 30-day mortality, 88.9% (16/18) occurred in individuals aged ≥65 years. The 30-day mortality rate was 1.2% (2/172) for those aged ≤65 years and 9.3% (16/172) for those aged ≥65 years. There was an association between 30-day mortality and long-term facility residence (χ^2^ *P* <0.001); 25% (9/36) of patients residing in a long-term facility died within 30 days.

**Table 2a:** Mortality based on principal manifestation.

Principal manifestation	Cases (n)	30-day mortality n (%)
Skin and soft tissue	110	7 (6.4)
Bone and joint	58	1 (1.7)
Central nervous system	3	0
Urosepsis	27	2 (7.4)
Gynaecological	64	0
Pneumonia	36	2 (5.6)
Infective endocarditis	7	1 (14.3)
Intra-abdominal	6	0
Sepsis	33	5 (15.2)
Total	344	18 (5.2)

ICU requirement based on principal manifestation is depicted in Table 2b. There was an association of principal manifestation with ICU requirement (χ^2^ *P* = 0.0003), particularly for IE (57.1%), and with risk factors such as cardiac disease (*P* = 0.003) and obesity (*P* = 0.010).

**Table 2b:** ICU requirement based on principal manifestation.

Principal manifestation	Cases (n)	Required ICU - n (%)
Skin and soft tissue	107	17 (15.9)
Bone and joint	56	15 (26.8)
Central nervous system	3	1 (33.3)
Urosepsis	26	2 (7.7)
Gynaecological	62	2 (3.2)
Pneumonia	35	8 (22.9)
Infective endocarditis	7	4 (57.1)
Intra-abdominal	5	2 (40.0)
Sepsis (without focus)	32	2 (6.3)
Totals^a^	333	53 (15.9)

ICU = intensive care unit.

a Missing data for ICU requirements; n = 11 were excluded from this analysis.

There was an association between older age (using median age) and pneumonia, SSTIs, sepsis, and urosepsis manifestations (*P* <0.001), with median ages of 79, 73, 70, and 75 years, respectively. Patients with gynaecological infections had the lowest median age of 31 years.

### *Investigations*

Of all cases, 297 had follow-up blood cultures within 28 days. The median time to the first negative blood culture was 2 days (range 0-20). There was no negative blood culture for 13.6% of isolates. There was an association between repeated blood cultures and 7- and 30-day mortality rates. The 7- and 30-day mortality rates for those who had no repeated blood cultures were 16.7% and 19.0%, respectively, as opposed to 0.3% and 3.3%, respectively, for those who had repeated cultures. Both results were statistically significant (*P* <0.005).

### *Imaging*

Imaging data were available for 329 cases, with TTE performed in 42.7%, CT in 51.5%, TOE in 7.3%, MRI in 19.2%, nuclear bone scan in 7%, gallium scan in 2.6%, and PET in 0.6%. CTs were performed most frequently in cases with IE (77.8%) and BJIs (69.0%) and least frequently in gynaecological infections (9.4%). MRIs were most frequently performed in BJIs (51.7%, 30/58). TTEs were performed in 88.9% of the total cases of IE (one patient declined the procedure), while TOEs were performed in 66.6%. A nuclear medicine bone scan was performed in 20.7% (12/58) of the total BJIs, accounting for 50% of the total cases that had a bone scan. BJIs were diagnosed in 88.9% (8/9) of patients who underwent gallium scans. Both patients who underwent PET scans also had SSTIs.

### *Antibiotics*

**Figure 3:**
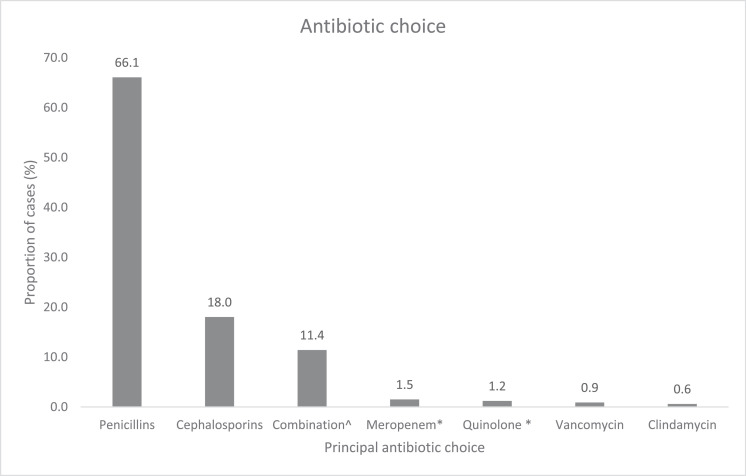
Principal antibiotic choice. Column graph depicting proportion of each antibiotic choice, represented in antibiotic class or combinations, as appropriate. Total n = 333. Excluded: unknown treatment n = 9, no antibiotic treatment n = 2. ^Refers to penicillin/cephalosporin + macrolide/vancomycin/gentamicin.

The principal antibiotic therapies according to antibiotic class are shown in Figure 3. A total of 332 patients were included in our antibiotic therapy analysis (n = 3, no antibiotic treatment; n = 9, missing data). Penicillin (66.1%) and cephalosporins (18.0%) were the most frequently used antibiotics. Penicillins or cephalosporins were used in combination therapies (11.4%), including macrolides (n = 11), vancomycin (n = 8), and gentamicin (n = 4). Allergies to penicillin (9.9%) and cephalosporins (2.3%) were recorded.

Shorter total antibiotic treatment durations were used for sepsis, pneumonia, and gynaecological manifestations, with a median of 14 days for each, whilst longer durations were used for CNS infections, BJIs, and IE, with medians of 85, 45, and 42.5 days, respectively.

## Discussion

There is increased morbidity and mortality associated with GBS bacteraemia, particularly among those aged ≥65 years or residing in a long-term care facility. In this study, we characterised the main presentations of GBS infections, including SSTIs, gynaecological manifestations, and BJIs, as well as the most common antibiotic therapy class, penicillin.

The median age at admission of 65 years [2] and the higher 30-day mortality rate for patients aged ≥65 years (*P* = 0.0007) reflect the findings of other studies [9,10]. Overall, there were more cases of GBS in women than in men in our study. However, among non-pregnant adults, only 47.6% were female, a proportion also supported by other studies [2,9]. We were unable to draw comparisons between Indigenous and non-Indigenous patients because of the small sample size of Indigenous patients in our study. Overall, both the rates of ICU admission [2,12] (15.9%) and 30-day mortality (5.2%) were found to be lower than those reported in similar studies, including those from Belgium (21,7%, 9.4%), Thailand (unknown, 11%), Taiwan (unknown, 20.2%), and the United States (27.3%, 5.7%) [2,9,12,13].

GBS rates appear to be increased in patients with comorbidities, as suggested in other studies [2]. The overall rate of diabetes in our study population (43.9%) was similar to those reported in other studies from both Australia and around the world [8,13]. We found that 83.1% of patients had at least one risk factor, a proportion lower than that reported in other studies [9,13].

SSTIs were determined to be the most frequent principal manifestation, which is comparable to the results of other studies [2,4,9,13,14]. There was an association between SSTI as a principal manifestation and diabetes (*P* <0.001). A 2018 study reported an association between presence of the risk factors diabetes mellitus and obesity and increased likelihood of invasive GBS disease, resultant osteomyelitis, and/or SSTI [15]. Similarly, we found that 44.4% of those with diabetes had SSTIs and 44.4% of those with obesity had SSTIs. A finding noted in another Australian study was that SSTIs and BJIs occasionally presented concurrently [8]. For example, in diabetic soft tissue infections resulting from or co-occurring with osteomyelitis, we found SSTIs concurrently with BJIs in 60.5% of the total BJI cases.

Pregnancy-related infections, which represented 16.3% of cases, may have increased the risk of GBS infection in the patients’ infants or foetuses [16]. Currently, GBS screening is not universally performed in Australia [17], but given the prevalence of pregnancy-related GBS disease, future revision and research could be warranted. A 2019 study found that a hexavalent vaccine could be promising for protecting neonates and their mothers with positive antenatal GBS screening results, as it covers six serotypes responsible for 98% of perinatal infections [18]. This vaccine is currently in phase II trials [19]. The same vaccine could be useful in protecting adults from GBS infection, as 95% of the serotypes responsible for adult infections are covered by this hexavalent vaccine [12]. Jones et al*.* (2022) reported that up to 15% of GBS isolates in their study in New South Wales would not be covered by this hexavalent vaccine [5]. This is perhaps due to the relative prominence of capsular genotypes VI-VIII in Asia [5], to which Australia is relatively close. A cost-benefit analysis may need to be performed before considering a vaccination strategy, with particular consideration given to high-risk populations.

Sepsis (or sepsis syndrome) was associated with the highest 7-day mortality rate. This was perhaps due to death or palliative measures undertaken before a formal diagnosis could be made. In our study, patients with IE and sepsis had the highest 30-day mortality rates (Fisher's exact test, *P* = 0.0459) (Table 2a). However, patients with pneumonia were found to have the highest 30-day mortality rate (39.1%) in a study from Thailand (2021) [9].

Definitive antibiotic choices in our study differed from those reported by Phoompoung et al. [9]. For instance, ceftriaxone (26.4%) was more commonly used than benzylpenicillin (12.9%) in their study, whereas our study found rates of 14.0% and 46.8%, respectively, with benzylpenicillin also being the most prescribed targeted antibiotic therapy. Furthermore, no cases of penicillin resistance were detected in our study. This is consistent with the results of antibiotic susceptibility testing from a 2022 study also conducted in New South Wales, Australia, which showed 100% susceptibility of invasive GBS isolates to penicillin [5]. These findings are consistent with those of international studies from Europe, Asia, and the Middle East [7,12,14].

### *Strengths and limitations*

Our study had a relatively large sample size and used a thorough manual data collection process. However, as the data relied solely on eMR reporting, they may have been incomplete because of under-reporting or missing medical record entries, particularly in the years before the widespread implementation of eMR (which was observed for pre-2017 data). The imaging choice and antibiotic duration were novel analyses included in our study. However, data for repeated blood cultures and imaging performed at private facilities or outpatient services were not available for our records, which could indicate potential underreporting of these investigations.

Cardiac disease was not categorised into ischaemic, valvular, and arrhythmogenic subtypes, which could have provided further stratification of the prevalence of particular risk factors for IE. Disease attributed to devices such as artificial valves and permanent pacemakers was recorded, although these numbers were too small for a meaningful analysis.

Using principal manifestations to analyse mortality, LOS, ICU requirement, and management choices simplifies what may be a complex clinical scenario with multiple foci of infection and manifestations. The use of imaging and antibiotics may have been affected by the presence of additional manifestations. However, owing to the heterogeneity of these additional manifestations, we were unable to account for them. We found that only 6.1% of the TTEs performed resulted in the diagnosis of IE, suggesting the need to refine the criteria on the basis of the pretest probability of IE and according to the modified 2023 Duke-International Society of Cardiovascular Infectious Diseases IE criteria [11]; however, there may have been other clinical indications for imaging.

Regarding the outcomes of ICU admission, such as mortality and LOS, there may have been contributory causes other than GBS bacteraemia. We did not explore the cause of death or reasons for ICU admission in our study. Patients may have developed polymicrobial infections after the initial blood culture, influencing the choice of antibiotics. Surgical or procedural interventions were not reviewed or considered when assessing antibiotic choice or duration.

## Conclusion

There is increased morbidity and mortality associated with GBS bacteraemia, especially among older adults. Known risk factors such as diabetes, obesity, and cardiac disease were found to be highly prevalent in those who had GBS bacteraemia. However, further studies and analyses are needed to determine the risk that these conditions pose for acquiring GBS bacteraemia. A substantial proportion of GBS bacteraemia cases were attributable to pregnancy-related infection. Benzylpenicillin was the mainstay therapy. The burden of the disease necessitates more research for prevention, especially for those at increased risk.

## Declarations of competing interest

The authors have no competing interests to declare.
